# Comparison of HIV Prevalence Estimates for Zimbabwe from Antenatal Clinic Surveillance (2006) and the 2005–06 Zimbabwe Demographic and Health Survey

**DOI:** 10.1371/journal.pone.0013819

**Published:** 2010-11-03

**Authors:** Elizabeth Gonese, Janet Dzangare, Simon Gregson, Nicholas Jonga, Owen Mugurungi, Vinod Mishra

**Affiliations:** 1 AIDS and TB Unit, Ministry of Health and Child Welfare, Harare, Zimbabwe; 2 Biomedical Research and Training Institute, Harare, Zimbabwe; 3 Imperial College London, London, United Kingdom; 4 Central Statistics Office, Harare, Zimbabwe; 5 ICF Macro, Calverton, Maryland, United States of America; University of Stellenbosch, South Africa

## Abstract

**Objective:**

To assess whether HIV surveillance data from pregnant women attending antenatal care (ANC) clinics in Zimbabwe represent infection levels in the general population.

**Methods:**

HIV prevalence estimates from ANC surveillance sites in 2006 were compared with estimates from the corresponding Zimbabwe Demographic and Health Survey 2005–06 (ZDHS) clusters using geographic information systems.

**Results:**

The ANC HIV prevalence estimate (17.9%, 95% CI 17.0%–18.8%) was similar to the ZDHS estimates for all men and women aged 15–49 years (18.1%, 16.9%–18.8%), for pregnant women (17.5%, 13.9%–21.9%), and for ANC attendees living within 30 km of ANC surveillance sites (19.9%, 17.1%–22.8%). However, the ANC surveillance estimate (17.9%) was lower than the ZDHS estimates for all women (21.1%, 19.7%–22.6%) and for women living within 30 km catchment areas of ANC surveillance sites (20.9%, 19.4%–22.3%). HIV prevalence in ANC sites classified as urban and rural was significantly lower than in sites classified as “other”.

**Conclusions:**

Periodic population surveys can be used to validate ANC surveillance estimates. In Zimbabwe, ANC surveillance provides reliable estimates of HIV prevalence among men and women aged 15–49 years in the general population. Three classifications of ANC sites (rural/urban/other) should be used when generating national HIV estimates.

## Introduction

Accurate HIV prevalence data are critical for countries in southern Africa faced with very high HIV-related disease burdens and limited resources. These data are required for monitoring the progress of the HIV epidemic, planning for HIV prevention and care and treatment programs, and assessing the impacts of interventions. The main source of HIV prevalence data is antenatal clinic (ANC) surveillance among pregnant women attending for antenatal care in selected health facilities. These data have been used to provide information on HIV prevalence levels and trends, including estimates for the general population derived using mathematical models [1]–[3].

The advantages and shortcomings of ANC data in representing the general population have been documented [4], [5]. The main advantages include the accessibility of populations and the low cost of data collection. However, lack of universal coverage of ANC services in developing countries and exclusion of men and non-pregnant women tend to make these data less representative of the general population. To obtain up-to-date and accurate data on HIV prevalence, countries have begun implementing HIV testing in population-based Demographic and Health Surveys (DHS) and AIDS Indicator Surveys (AIS). These surveys provide nationally representative estimates of HIV prevalence in the general population and have the advantage of linking socio-demographic and behavioral data to the HIV serostatus of individuals [6]. However, estimates derived from these surveys can be affected by bias, due to non-response and exclusion of non-household-based populations, and the surveys are too expensive to conduct on an annual or biannual basis.

A comparison of HIV prevalence estimates from population-based surveys to those from ANC surveillance in five Sub-Saharan African countries—Ethiopia, Kenya, Malawi, Tanzania, and Uganda—showed that population survey estimates were lower than ANC estimates in four of the five countries [7], [8]. In Uganda, where the HIV epidemic has stabilised, the estimates were similar from both sources. In the multi-country analysis, younger women (age 15–24) sampled in the ANC surveillance catchment areas in the population-based surveys had a lower HIV prevalence than those in the ANC surveillance surveys. The opposite trend was observed for older women (age 25–49). A similar pattern has been observed in local studies in Zimbabwe [9]. Variations in HIV prevalence were also noted for the different residential classifications (urban and rural).

These comparisons provide insight into the potential biases of the different data sources. Researchers concluded that the two data sources (population-based surveys and ANC surveillance surveys) are complementary and that caution needs to be exercised in interpreting HIV prevalence data [7].

Zimbabwe has conducted ANC surveys biannually in 19 consistent sites since 2000. The 2006 round coincided with the 2005–06 Zimbabwe Demographic and Health Survey (ZDHS—the first national population-based survey that included HIV testing. The extent to which HIV prevalence data from the ANC surveillance surveys reflect prevalence in the general population has not previously been assessed at the national level in Zimbabwe. Therefore, this study compares HIV prevalence estimates from the 2006 ANC surveillance survey with estimates from the 2005–06 ZDHS for women living in the sampled clusters within the catchment areas of the ANC surveillance sites.

The 2005–06 ZDHS data have been used previously to calibrate the 2007 HIV national estimates in Zimbabwe. The HIV estimation process for Zimbabwe has been unique in that, in addition to the usual two residential classifications—urban and rural—a third classification of “other”, derived from the classifications employed in the national census, is used. ANC sentinel sites classified as “other” are characterized by high labor and circulatory migration and include growth points, commercial farming areas, mining areas, and border towns. It is believed that the epidemiology of HIV in these communities is different than that in either urban or rural settings [9]. A scientific audit to determine the value of the “other” residential classification at the national level has not been conducted. This analysis will therefore also explore the differences in HIV prevalence by the three different classifications (rural, urban, and other) in ANC surveillance data compared with the ZDHS clusters within a 30 km catchment area of each ANC surveillance site.

## Methods

### Ethics statements

“Please be advised that the Medical Research Council of Zimbabwe has reviewed and approved your application to conduct your study entitled ‘Routine HIV antenatal clinic surveillance among pregnant women. Supplementary studies, HIV drug resistance threshold survey, HIV incidence.’ Approval number MRCZ/A/1284.”

“Please be advised that the Medical Research Council of Zimbabwe has reviewed and approved your application to conduct the study entitled ‘Anaemia and HIV testing in the Zimbabwe Demographic and Health Survey 2005.’ This approval includes approval of the following: informed consent form; Demographic and Health Survey Women's Questionnaire; Demographic and Heath Survey Man's Questionnaire; Demographic and Health Survey Household Questionnaire. Approval number MRCZ/A/1188/11.”

### The 2006 ANC Surveillance Survey

A total of 19 sentinel sites contributed to the 2006 ANC surveillance survey. Whilst the national surveillance system is not designed to provide fully representative national estimates for Zimbabwe, the 19 ANC sentinel sites were purposively chosen from urban, rural and ‘other’ areas in each province to provide a roughly representative picture of levels and trends in HIV prevalence for the country. Three sites in major cities were over-sampled in order to give a larger sample size in the 15–24 age group in urban areas, which could be used as a proxy for HIV incidence [10]. Pregnant women presenting for the first time with their current pregnancy at the participating ANC sites during the survey period were enrolled in the study. A total of 7,249 ANC attendees were tested in an anonymous unlinked HIV sero-survey. A minimum data set extracted from antenatal clinic booking cards was used to fill in the 2006 ANC survey form. More details about the ANC survey are available in the main survey report [11].

### The 2005**–**06 ZDHS

In the 2005–06 ZDHS the sample was selected in two stages, with enumeration areas (EAs) as the first-stage and households as the second-stage sampling units. In total, 1,200 enumeration areas were selected with probability proportional to size (PPS), the size being the number of households enumerated in the 2002 Census. The list of households obtained was used as the frame for the second-stage systematic probability selection of households. The listing excluded people living in institutions (army barracks, hospitals, police camps, boarding schools, etc.) and the homeless.

All women age 15–49 and men age 15–54 who were either permanent residents of the sampled households or visitors present in the household on the night before the survey were eligible to be interviewed and to give consent for blood draw for anemia and HIV testing.

### Geographic Information Systems (GIS) Method

A GIS-based method was used to identify the ZDHS clusters that were located within a 30 km radius of the nearest ANC sentinel site. Although Zimbabwe endeavours to provide primary health care services within a 10 km radius, a wider radius was used since ANC sentinel sites often have a wider geographic coverage, because there is a good road network and people tend to seek care at higher-level health institutions. Additionally, primary health care facilities do not offer all mother and child health services, and differences in user fees can widen the geographic catchment areas for some sites.

Each of the 19 ANC sentinel sites was matched to the nearest ZDHS enumeration area using geo-reference codes in ArcView 9.1 [12]. In each case, a ZDHS enumeration area was found within 30 km of the ANC.

Of 6,947 women interviewed and tested for HIV in the 2005–06 ZDHS, 2,943 (42%) lived in clusters located within 30 km of one of the 19 ANC sites. We compared the ANC surveillance survey estimates of HIV prevalence with the estimates for all men and women (combined and separately) included in the ZDHS, women living in 30 km ANC catchment areas, and women living in 30 km ANC catchment areas who attended ANC for their last birth. In the initial analyses (Tables 1–3), ZDHS clusters and ANC sites were distinguished as urban and rural using the ZDHS classification. In Table 4, ZDHS clusters that were within 30 km radius of ANC surveillance sites classified as “other” were recoded from “urban” or “rural” to “other” to allow comparisons of HIV prevalence estimates for each of the three residential strata.

**Table 1:** Sample distributions of women (aged 15–49 yrs) included in the 2006 ANC surveillance survey and the 2005–06 Zimbabwe Demographic and Health Survey, by selected characteristics.

	ANC^3^		ZDHS					
	All women (15–49)		All women (15–49) interviewed and tested for HIV		Women in 30 km ANC catchment areas^1^		Women in 30 km ANC catchment areas who attended ANC for last birth^2^	
	%	N	%	N	%	N	%	N
**Total**	100.0	7202	100.0	6947	100.0	2943	100.0	777
**Age group**								
15–24	58.7	4236	46.1	3200	48.2	1417	44.9	349
25–34	35.3	2547	30.3	2105	29.2	860	45.3	352
35–49	6.0	435	23.6	1642	22.6	666	9.8	76
**Residence**								
Urban	52.4	3768	38.4	2670	71.8	2113	65.1	506
Rural	47.7	3430	61.6	4277	28.2	830	34.9	271
**Education**								
None	0.9	66	4.3	301	2.1	61	*	11
Primary	21.3	1531	32.6	2263	21.2	623	19.7	153
Secondary/higher	77.8	5596	63.1	4383	76.8	2259	78.9	613
**Work status**								
Not working	85.5	6158	63.4	4406	63.4	1866	67.3	523
Working	14.5	1048	36.6	2541	36.6	1077	32.7	254
**Marital status**								
Never married	5.0	354	26.6	1846	32.5	957	(5.7)	44
Married	94.0	6699	58.0	4027	51.9	1527	83.5	649
Divorced/separated/ Widowed	1.0	71	15.5	1074	15.6	459	10.8	84
**Number of living children** 4								
0	47.5	3414	30.0	2086	35.3	1040	40.03	311
1–2	41.2	2960	37.3	2590	38.2	1123	44.14	343
3–4	9.6	688	20.2	1401	18.2	536	11.71	91
5+	1.8	130	12.5	871	8.3	244	(4.1)	32

1 Women aged 15–49 yrs interviewed and tested by the ZDHS who lived within 30 km of the nearest ANC site.

2 Women aged 15–49 yrs interviewed and tested by the ZDHS who lived within 30 km of the nearest ANC site and received ANC for their last birth in the previous three years.

3 Ns for individual categories may not add up to the total due to missing information.

4 Number of living children for women in the ZDHS sample who live within an ANC catchment area and attended ANC for the last birth has been adjusted to show parity at the time of the last ANC attendance (except for the most recent birth).

*0–24 unweighted case; () 25–49 unweighted cases.

**Table 2:** Comparison of HIV prevalence among women age 15–49 from ANC sentinel surveillance and among men and women aged 15–49 interviewed by the ZDHS, by women's pregnancy status, recent birth experience, and receiving antenatal care for last birth, 2005–06.

	ANC			ZDHS					
	All women (15–49)			All women (15–49)^1^			Women in 30 km ANC catchment areas^2^		
	%	95%CI	N	%	95%CI	N	%	95%CI	N
**Total**	17.9	17.0–18.8	7202	21.1	19.7–22.6	6947	20.9	19.4–22.3	2943
**Currently pregnant**									
No		---	---	21.4	19.9–23.0	6473	21.3	19.8–22.8	2789
Yes		---	---	17.5	13.9–21.9	474	13.0	7.6–18.4	154
**Gave birth in past 3 years**									
No		---	---	21.5	20.1–23.0	4602	21.0	19.3–22.7	2136
Yes		---	---	20.3	18.2–22.7	2345	20.4	17.7–23.2	807
**Attended ANC for last birth (among women who gave birth in last 3 years)**									
No		---	---	26.0	17.8–36.2	97	(33.3)	15.4–51.2	30
Yes		---	---	20.1	17.9–22.5	2248	19.9	17.1–22.8	777

1 ZDHS HIV prevalence estimate for all men age 15–49 is 14.5% (CI 13.2–15.9), and for all men and women age 15–49 is 18.1% (CI 16.9–18.8).

2 Women 15–49 interviewed and tested by the ZDHS who live in a community within 30 km from the nearest ANC site.

( ): 25–49 unweighted cases.

**Table 3:** Comparison of HIV prevalence in women aged 15–49 years: ANC sentinel surveillance versus ZDHS, by selected background characteristics, 2005–06.

	ANC			ZDHS								
	All women (15–49)			All women (15–49)			Women in 30 km ANC catchment areas^1^			Women in 30 km ANC catchment areas who attended ANC for last birth^2^		
	%	95%CI	N	%	95%CI	N	%	95%CI	N	%	95%CI	N
**Total**	17.9	17.0–18.8	7202	21.1	19.7–22.6	6947	20.9	19.4–22.3	2943	19.9	17.1–22.8	777
**Age group**												
15–24	13.3	12.2–14.3	4224	11.0	9.8–12.3	3200	10.7	9.0–12.3	1417	14.0	10.4–17.7	349
25–34	25.0	23.3–26.6	2545	31.8	29.0–34.8	2105	31.6	28.5–34.7	860	26.7	22.1–31.3	352
35–49	21.7	17.8–25.6	433	27.1	24.6–29.7	1642	28.7	25.2–32.1	666	15.8	7.4–24.2	76
**Residence**												
Urban	18.6	17.3–20.0	3422	21.6	19.8–23.6	2670	20.9	19.1–22.6	2113	20.9	17.4–24.5	506
Rural	17.2	16.0–18.4	3760	20.8	18.8–23.0	4277	20.8	18.1–23.6	830	18.1	13.5–22.7	271
**Education**												
None	16.7	7.4–25.9	66	20.0	14.4–27.0	301	26.2	14.9–37.6	61	*	*	11
Primary	18.9	17.0–20.9	1526	22.4	19.8–25.3	2263	24.6	21.2–27.9	623	17.0	11.0–23.0	153
Secondary/higher	17.6	16.6–18.6	5585	20.5	19.0–22.2	4383	19.7	18.1–21.3	2259	21.0	17.8–24.3	613
**Work status**												
Not working	17.4	16.4–18.3	6143	19.3	17.8–21.0	4406	19.3	17.6–21.1	1866	20.1	16.6–23.5	523
Working	20.8	18.4–23.3	1047	24.2	22.1–26.4	2541	23.5	21.0–26.0	1077	19.7	14.8–24.6	254
**Marital status**												
Never married	21.2	17.0–25.5	353	8.4	7.2–9.8	1846	9.3	7.5–11.1	957	(25.0)	11.7–38.3	44
Married	17.5	16.6–18.4	6684	20.2	18.7–21.9	4027	21.2	19.2–23.3	1527	18.2	15.2–21.2	649
Divorced/separated/ Widowed	42.3	30.5–54.0	71	46.3	42.1–50.5	1074	43.8	39.2–48.3	459	31.0	20.9–41.0	84
**Number of living children** 3												
0	13.0	11.9–14.1	3405	10.0	8.6–11.7	2086	10.3	8.4–12.1	1040	17.4	13.1–21.6	311
1–2	22.4	20.9–23.9	2956	26.8	24.7–29.1	2590	26.2	23.6–28.8	1123	21.3	16.9–25.6	343
3–4	23.0	19.9–26.2	686	28.5	25.7–31.4	1401	30.4	26.5–34.3	536	25.3	16.2–34.4	91
5+	15.5	9.2–21.8	129	18.9	15.7–22.6	871	20.5	15.4–25.6	244	(15.6)	2.3–28.9	32

1 Women aged 15–49 yrs interviewed and tested by the ZDHS who live within 30 km of the nearest ANC site.

2 Women aged 15–49 yrs interviewed and tested by the ZDHS who live within 30 km of the nearest ANC site and who received ANC for their last birth in the previous 3 years.

3 Number of living children for women in the ZDHS sample who live within an ANC catchment area and attended ANC for the last birth adjusted to show parity at the time of the last ANC attendance (excluding the most recent birth).

*: 0–24 unweighted case; (): 25–49 unweighted cases.

### Statistical Analysis

The comparisons in HIV prevalence estimates were made by selected demographic and socioeconomic characteristics of women available in both the ANC surveillance survey and the ZDHS. These included broad age groups, educational status, work status, marital status, number of living children, and urban/rural residence. ZDHS estimates were also tabulated for women by current pregnancy status, experience of birth in past three years, and whether attended ANC for last birth in past three years.

No reliable information was available for the population sizes in the ANC catchment areas or on the representativeness of the ANC surveillance sites. Therefore, we did not have appropriate weighting factors for the estimates based on the 2006 ANC surveillance survey or for women in the 2005–06 ZDHS living in the ANC catchment areas, and comparisons were made using un-weighted estimates. However, the estimates for all women in the ZDHS were appropriately weighted to provide comparisons with nationally-representative estimates.

STATA SE10.1 statistical software [13] was used to recode variables and generate HIV prevalence estimates and 95% confidence intervals (CI) for both the 2006 ANC surveillance survey and the 2005–06 ZDHS datasets.

## Results

In total, 7,494 women (76% of those eligible) and 5,555 men (63% of those eligible) had a valid HIV test result in the 2005–06 ZDHS. Of the women participating in the ZDHS, 2,943 lived within 30 km of an ANC surveillance site. Of these women, 777 had attended ANC for their last birth in the previous three years. A total of 7,202 pregnant women participated in the anonymous unlinked sero-survey conducted in the 19 ANC sentinel sites in 2006.

A comparison of women included in the 2006 ANC surveillance survey and those in the 2005–06 ZDHS reveals major differences in their characteristics. Women in the ANC survey were younger, had fewer children, were more educated and were more likely to be unemployed, married, and living in urban areas (Table 1). The characteristics of the women in the ANC survey were more similar to those of women interviewed in the ZDHS who lived in the 30 km catchment areas of the ANC surveillance sites—particularly so when the sample was further restricted to include only women who attended ANC for their last birth in the past three years. However, women in the ZDHS who lived in the 30 km ANC catchment areas, had had a birth in the last three years and reported attending for ANC for their most recent birth were somewhat older than those in the ANC survey and were more likely to live in urban areas, to be working, and to be divorced, separated or widowed (Table 2). This seems most likely to have resulted from our selection of a somewhat arbitrary 30 km radius for the catchment areas for the ANC sites which could have caused the higher proportion of urban women in the ZDHS sample.

The un-weighted pooled ANC sentinel surveillance HIV prevalence estimate for women (17.9%, CI 17.0%–18.8%) is similar to that for all men and women age 15–49 (18.1%, 16.9%–18.8%) in the ZDHS. The ANC estimate is significantly lower than the ZDHS estimates for all women (21.1%, 19.7%–22.6%) and women living in the 30 km ANC catchment areas (20.9%, 19.4%–22.3%), and higher than that for men age 15–49 (14.5%, 13.2%–15.9%). It is also lower than the ZDHS estimate for women who reported a birth in the last three years and attended ANC for their most recent birth (19.9%, 17.1%–22.8%). However, HIV prevalence has been declining in Zimbabwe and prevalence amongst ANC attendees was slightly higher one year earlier - the average of the ANC estimates for 2004 (21.3%) and 2006 (17.9%) is 19.6% - when the latter group of women would have been attending ANC. In addition, the older ages of the recently pregnant women in the ZDHS sample would be expected to raise HIV prevalence somewhat but also to increase the proportion of women who are at the more advanced stages of infection where HIV-associated sub-fertility is generally most severe [14].

HIV prevalence in the ANC survey was higher among younger women (age 15–24) and lower among older women (age 25–49) than in the corresponding age-groups of women in the ZDHS (Table 3). HIV prevalence among women tested in the ANC survey was lower than in women tested in the ZDHS for all socio-economic sub-groups except those who had never been married and those with no living children. However, these differences disappeared when the comparison was restricted to women in the ZDHS who lived in the 30 km ANC catchment areas, had had a birth in the last three years, and who reported attending for ANC for their most recent birth.

By residential classifications, HIV prevalence in the ANC survey was lowest in the rural areas (15.1%, CI 17.0%–18.8%), higher in the urban areas (17.8%, 16.5%–19.1%), and highest in the areas classified as “other” (23.3%, 21.1%–25.6%) (Table 4). When the ZDHS women in the 30 km catchment areas for the ANC sites were grouped according to the ANC site classification, the ANC survey estimates remained lower than the ZDHS estimates for the urban and rural classifications. In the “other” classification HIV prevalence in the 2006 ANC surveillance survey was higher than in the ZDHS. However, the ZDHS sample for “other” sites was small and were dominated by one site that had relatively low prevalence even in the ANC survey, and the difference was not statistically significant.

**Table 4:** Comparison of HIV prevalence in women aged 15–49: ANC sentinel surveillance versus ZDHS, by ANC surveillance site and site classification, 2005–06.

		ANC			ZDHS		
Province/ANC site	ANC site classification	All women (15–49)			Women in 30 km ANC catchment areas^1^		
		%	95%CI	N	%	95%CI	N
**Total**		**17.9**	17.0–18.8	7202	**20.9**	19.4–22.3	2943
**Rural**		**15.1**	13.7–16.5	2472	**21.6**	17.5–25.7	394
Binga District Hospital	Rural	7.6	4.8–10.4	344	*	*	17
Gutu Mission Hospital	Rural	17.3	13.5–21.1	387	*	*	16
Karanda Hospital	Rural	10.0	6.8–13.2	339	22.6	13.9–31.2	93
Murambinda Hospital	Rural	16.4	12.4–20.4	356	22.9	12.8–32.9	70
Musume Mission Hospital	Rural (Growth Point)	18.5	14.5–22.6	356	20.8	9.5–32.0	53
Mutoko District Hospital	Rural (Growth Point)	17.5	13.3–21.6	326	16.9	8.0–25.8	71
Sadza District Hospital	Rural	17.6	13.9–21.4	391	25.7	15.5–35.9	74
**Urban**		**17.8**	16.5–19.1	3388	**20.7**	19.0–22.3	2283
Bindura Chipadze Clinic	Urban	13.5	9.9–17.1	348	16.8	9.4–24.3	101
Chinotimba Clinic	Urban (Border Post)	25.5	20.8–30.2	337	18.0	7.0–29.0	50
Gwanda Provincial Hospital	Urban (Municipality)	24.7	20.0–29.4	328	25.9	14.2–37.5	58
Gweru Provincial Hospital	Urban (Municipality)	18.0	13.9–22.2	333	25.3	16.4–34.2	95
Kuwadzana Clinic	Urban (Municipality)	15.8	12.8–18.9	550	19.7	16.8–22.6	725
Nkulumane Clinic	Urban (Municipality)	18.0	14.9–21.1	590	19.9	16.9–23.0	672
Sakubva Clinic	Urban (Municipality)	14.5	10.7–18.3	331	18.9	12.2–25.7	132
St Mary's Clinic	Urban (Municipality)	15.4	12.4–18.4	571	23.3	19.4–27.3	450
**Other**		**23.3**	21.1–25.6	1342	**21.4**	16.5–26.4	266
Banket District Hospital	Other (Commercial farming)	24.9	20.2–29.6	329	29.2	17.9–40.6	65
Beitbridge District Hospital	Other (Border Post)	25.5	20.7–30.2	330	(23.7)	9.5–37.8	38
Chiredzi District Hospital	Other (Commercial farming)	20.3	16.0–24.6	345	19.0	12.5–25.5	142
Kadoma District Hospital	Other (Mining)	22.8	18.3–27.3	338	*	*	21

1 Women 15–49 interviewed and tested by the ZDHS who live in a community within 30 km from the nearest ANC site.

Urban, rural, and other designation is based on the classification of the ANC surveillance site attended (for ANC columns) or the nearest ANC surveillance site (for ZDHS columns).

*: 0–24 unweighted case; (): 25–49 unweighted cases.

## Discussion

The 2006 ANC surveillance estimate (17.9%, 95% CI 17.0%–18.8%) provides a good approximation to HIV prevalence among men and women in the general population measured in the 2005–06 ZDHS (18.1%, 16.9%–18.8%). This finding is consistent with findings from similar national comparisons in five sub-Saharan African countries [7] and from a number of earlier community studies [9], [15], [16], and supports UNAIDS recommendations that routine ANC surveillance data can be used to provide reliable national estimates of HIV prevalence in adults [17].

The ANC estimate understated HIV prevalence in women in the general population but overstated HIV prevalence in men. These results are also consistent with findings from the earlier studies [7]–[9]. In general, estimates based on pregnant women tend to overstate HIV prevalence among all women at young ages, due to selection for early sexual activity, and overstate prevalence at older ages, due to infertility and/or higher levels of contraceptive use among infected women [18]. The latter effect is typically stronger and results in net underestimates for women in the general population, as we observed in the current study. ANC surveys typically overestimate HIV prevalence in men because HIV prevalence is generally lower in men than in women aged 15–49 due to their older average ages at infection [19].

HIV prevalence in the ANC survey was slightly lower than in the ZDHS among women who attended for ANC for their last birth. However, this difference can be explained by the approximately one-year difference between the date of the ANC survey and the average date when women in the ZDHS most recently attended for antenatal care. Thus the study results indicate that, in countries where access to ANC services is nearly universal, it is possible to get a reliable estimate of HIV prevalence among pregnant women using ANC sero-surveys.

In most countries HIV prevalence is higher in urban areas than in rural areas [20], [21]. In the ANC survey in Zimbabwe, HIV prevalence was slightly higher in the urban areas (17.8%) than in the rural areas (15.1%) but was higher still in the areas classified as other (23.2%) which are characterised by high levels of circulatory labour migration. When the ZDHS data for all women living in the 30 km ANC site catchment areas were grouped according to the urban/rural/other ANC site classification, HIV prevalence was found to be similar in all three types of area. HIV prevalence was higher among the pregnant women tested in the ANC survey in the rural and urban areas but not in the other areas. This finding may be because the ZDHS estimate for the “other” classification was based on a relatively small and unrepresentative sample.

There are some limitations in this study that should be kept in mind when interpreting its findings. The 30 km radius around the ANC surveillance sites used in identifying matching ZDHS clusters may not reflect the true catchment areas for the individual ANC sites. The GPS coordinates of the ZDHS clusters were displaced to protect confidentiality of survey participants. However, this displacement was random and the results from individual ANC catchment areas were aggregated up to the national level, so any effect of such bias is expected to be small. The ZDHS sample may also be biased due to differential non-response in the survey and/or exclusion of population groups that do not live in households. An analysis of the effects of non-response and exclusion of non-household-based populations on national HIV prevalence estimates derived from household surveys in several countries found that this bias was generally small [22]. Finally, the small numbers of women in the ZDHS sample who lived in the catchment areas of the ANC surveillance sites and attended ANC for their last birth makes it difficult to interpret the differentials in prevalence for these women observed by urban, rural, and other site classification.

In conclusion, our comparison of HIV estimates finds that the ANC surveillance estimate compares well with the overall HIV prevalence estimate from the ZDHS population survey for all adults (men and women). This is despite important differences in the characteristics of the women who participated in the two surveys. The findings suggest that ANC surveillance provides reliable estimates of HIV prevalence among pregnant women attending ANC clinics and is a useful source of data for monitoring the HIV epidemic in Zimbabwe. At the same time, periodic sero-behavioral surveys, such as the ZDHS, that provide HIV prevalence data for representative samples of adults in the general population, can be helpful in validating ANC-based HIV estimates and in understanding the biases in ANC data.

In addition, they provide linked information on the characteristics and risk-taking and healthcare-seeking behaviors of infected and uninfected adults, which can aid the design of effective HIV programs. Finally, the much higher HIV prevalence seen at ANC sites classified as “other” suggests that Zimbabwe should continue to generate HIV estimates using the three classifications: rural, urban, and other.
